# Massive calcified solid pseudopapillary neoplasm of the pancreatic head

**DOI:** 10.18632/oncoscience.642

**Published:** 2026-02-07

**Authors:** Faten Limaiem, Mohamed Hajri

**Affiliations:** ^1^Department of Pathology, Hospital Mongi Slim La Marsa, La Marsa, Tunis, 2046, Tunisia; ^2^Department of Surgery, Hospital Mongi Slim La Marsa, La Marsa, Tunis, 2046,Tunisia

**Keywords:** solid pseudopapillary neoplasm, pancreas, surgery, pathology, immunohistochemistry

## Abstract

Solid pseudopapillary neoplasm (SPN) of the pancreas is an uncommon, low-grade malignant tumor, accounting for less than 3% of all exocrine pancreatic neoplasms. Although typically indolent, SPN poses significant diagnostic challenges and must be distinguished from other pancreatic tumors to guide appropriate management. We present the case of a 31-year-old woman with a two-year history of right upper abdominal pain which had recently worsened. Laboratory investigations, including tumor markers, were within normal limits. Imaging revealed a massive, lobulated pancreatic head tumor with solid, cystic, and calcified components. The mass was in close contact with the duodenum, the splenomesenteric confluence, and the right colic flexure, without evidence of invasion. The patient underwent a cephalic pancreaticoduodenectomy (Whipple procedure). Gross examination showed a well-encapsulated 12.5 × 9 × 8 cm mass with cystic degeneration, hemorrhage, and coarse calcifications. Histological examination revealed solid and pseudopapillary architecture with low mitotic activity and degenerative changes. Immunohistochemistry demonstrated positivity for β-catenin and CD10, confirming the diagnosis of SPN. Complete surgical resection is associated with an excellent prognosis, although rare aggressive behavior has been reported. This case underscores the diagnostic value of integrating imaging, histopathology, and immunohistochemistry. It also highlights the importance of considering SPN in the differential diagnosis of large, calcified pancreatic masses in young women.

## INTRODUCTION

Solid pseudopapillary neoplasm (SPN) of the pancreas is an uncommon, low-grade malignant tumor, accounting for approximately 0.9% to 2.7% of all exocrine pancreatic neoplasms [1]. Despite its rarity, SPN represents a significant diagnostic challenge in gastroenterology, oncology, and pathology. This challenge is mainly related to its heterogeneous imaging features and its overlap with other pancreatic tumors and cystic lesions [1–3]. Early and accurate differentiation is essential, as it directly guides surgical management and helps optimize patient outcomes. Definitive diagnosis relies on a combination of advanced imaging techniques, pathognomonic histologic patterns, and characteristic immunohistochemical profiles. After complete surgical resection, SPNs generally have an excellent prognosis. However, very large tumors with extensive calcification are uncommon, and their clinical, radiologic, and pathologic features remain poorly characterized. This limited evidence complicates understanding of their biological behavior and diagnostic evaluation. [1–3]. Herein, we report a case of a large, calcified SPN in a young woman. The aim of this report is to highlight the diagnostic challenges associated with this unusual presentation and to emphasize the pivotal role of integrating imaging findings with histopathology for accurate diagnosis.

## CASE PRESENTATION

### Patient history and presenting complaint

A 31-year-old woman, previously treated surgically for a left breast fibroadenoma, presented with right upper abdominal pain lasting two years. The pain had recently worsened, leading her to seek medical attention.

### Physical examination

On admission, the patient was in good general condition. Physical examination was unremarkable, with no palpable masses or abdominal tenderness.

### Imaging, laboratory findings

**Figure 1:**
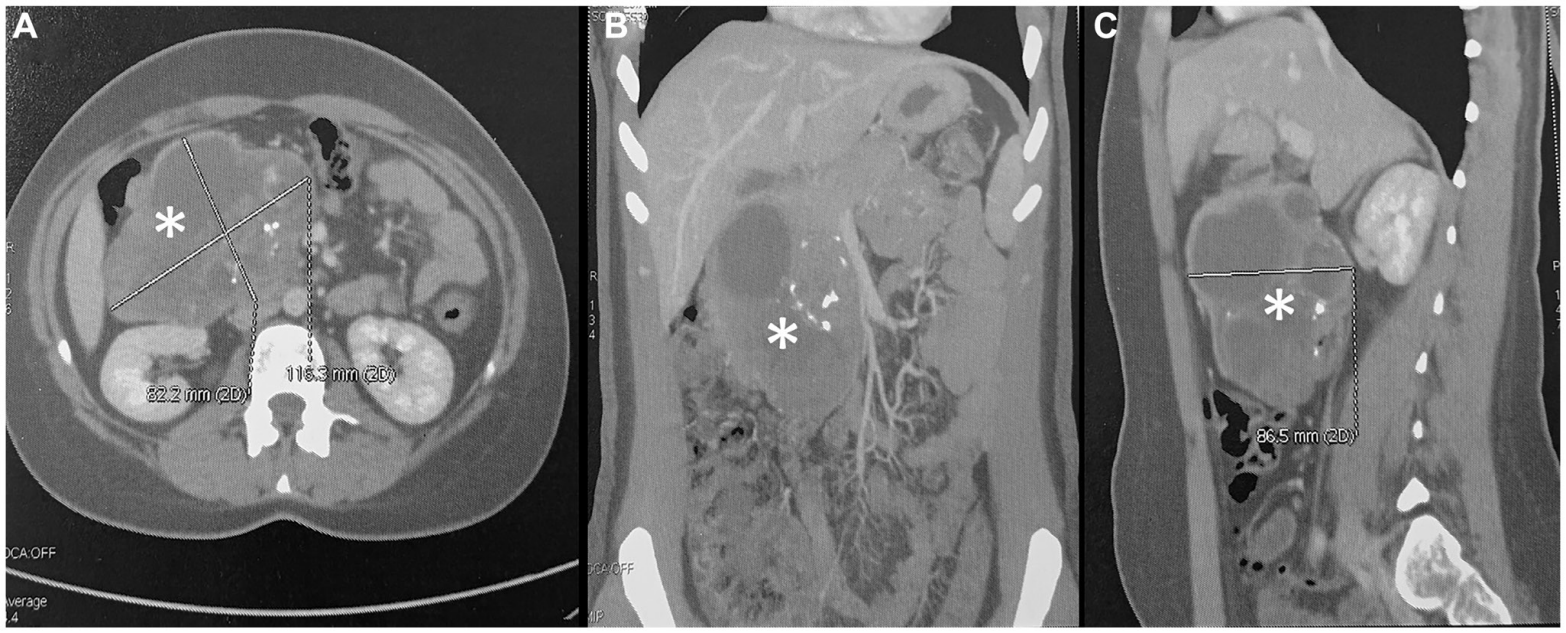
Contrast-enhanced abdominal CT of a large retroperitoneal mass. The well-defined mass exhibits heterogeneous enhancement with scattered calcifications. (**A**) Axial view shows the lesion (asterisk) measuring approximately 8.2 × 11.8 cm. (**B**, **C**) Coronal (B) and sagittal (C) reconstructions demonstrate the craniocaudal and anteroposterior (8.6 cm) extent of the mass, respectively, and its displacement of adjacent abdominal structures.

Laboratory tests, including tumor markers, were within normal limits. Carcinoembryonic antigen was less than 0.50 ng/mL, and carbohydrate antigen 19–9 was less than 3 U/mL. Abdominal ultrasonography revealed a large solid–cystic mass arising from the pancreas. Contrast-enhanced abdominopelvic computed tomography (CT) demonstrated a lobulated mass in the pancreatic head measuring 11.6 × 8.5 cm in axial dimensions and 7.2 cm in height. The lesion contained solid, cystic, and calcified components. It was in close contact with the duodenum, the splenomesenteric confluence, and the right colic flexure, without evidence of invasion (Figure 1). Magnetic resonance imaging (MRI) confirmed a large pancreatic head mass measuring 12 × 7.7 cm. The tumor showed mixed solid, cystic, and calcified components. It abutted the right colic flexure and the inferior vena cava, without biliary or pancreatic ductal dilatation. Overall, the imaging findings were most suggestive of a solid pseudopapillary neoplasm of the pancreas.

### Surgical management

The patient underwent a cephalic pancreaticoduodenectomy (Whipple procedure). The specimen was submitted for histological examination.

### Pathological findings

**Figure 2:**
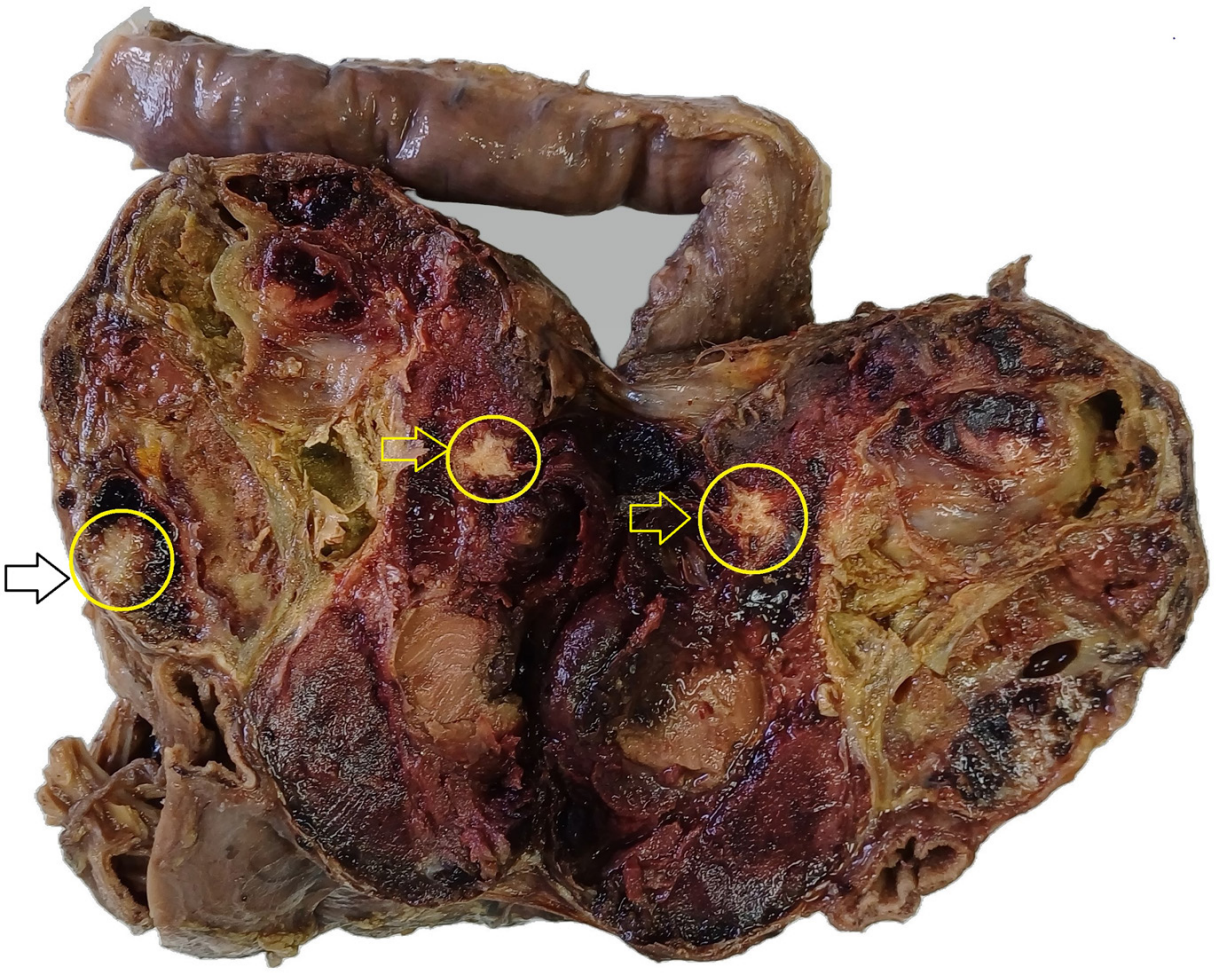
Gross photograph of the duodenopancreatectomy specimen. A well-demarcated, solid-cystic mass (12.5 × 9 × 8 cm) is present in the pancreatic head. The cut surface shows a heterogeneous appearance with areas of cystic degeneration, hemorrhage, and coarse calcifications (arrows).

**Figure 3:**
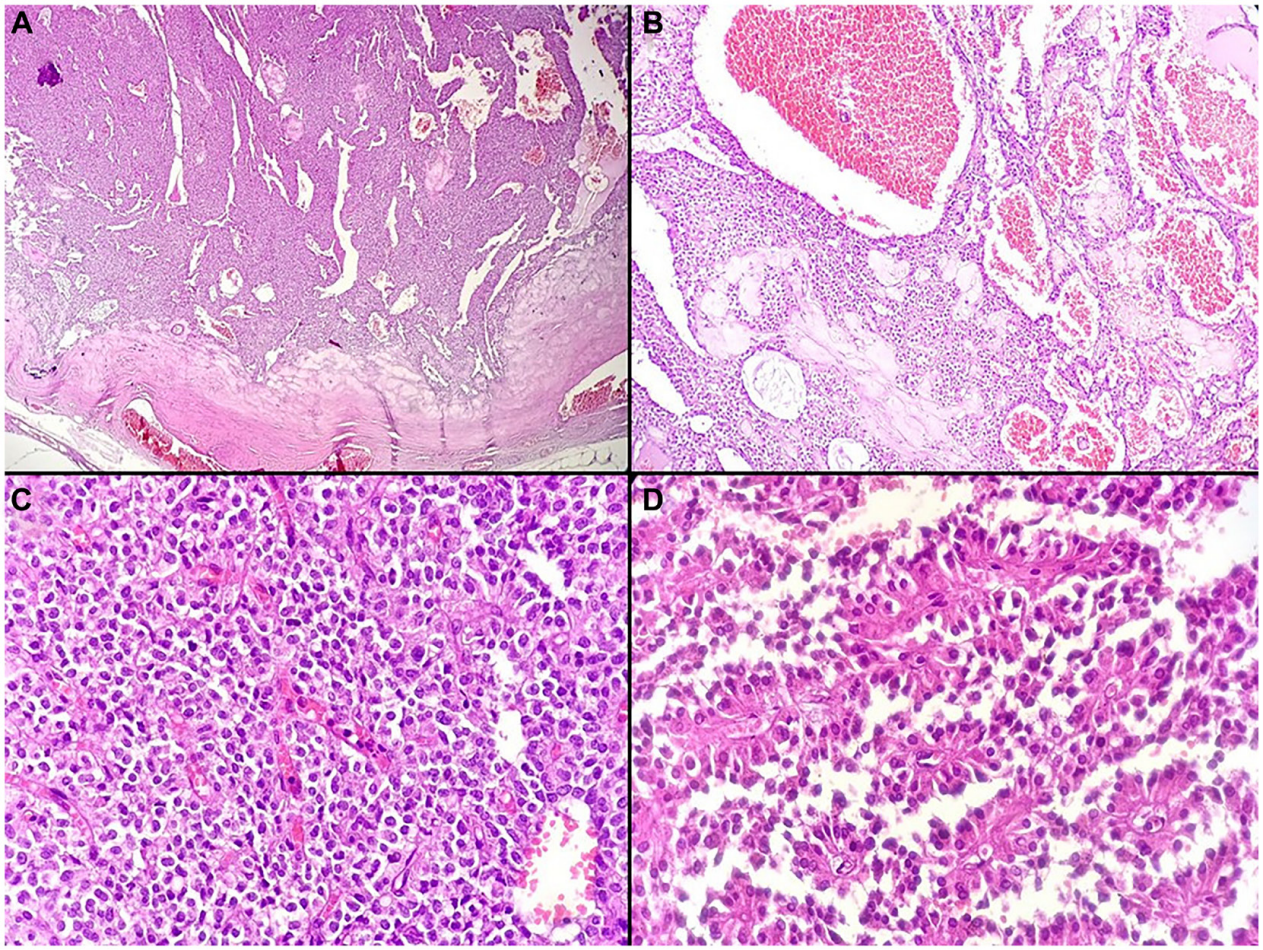
Histopathological features of the solid pseudopapillary neoplasm. (**A**) Low-power view showing a well-encapsulated neoplasm with solid and pseudopapillary areas (H&E, ×40). (**B**) Tumor with a prominent vascular network, including multiple congested, dilated capillaries (H&E, ×100). (**C**) Solid areas composed of uniform cells separated by delicate capillary-sized vessels (H&E, ×400). (**D**) Pseudopapillae formed by tumor cell detachment around fibrovascular cores, creating rosette-like structures (H&E, ×400).

**Figure 4:**
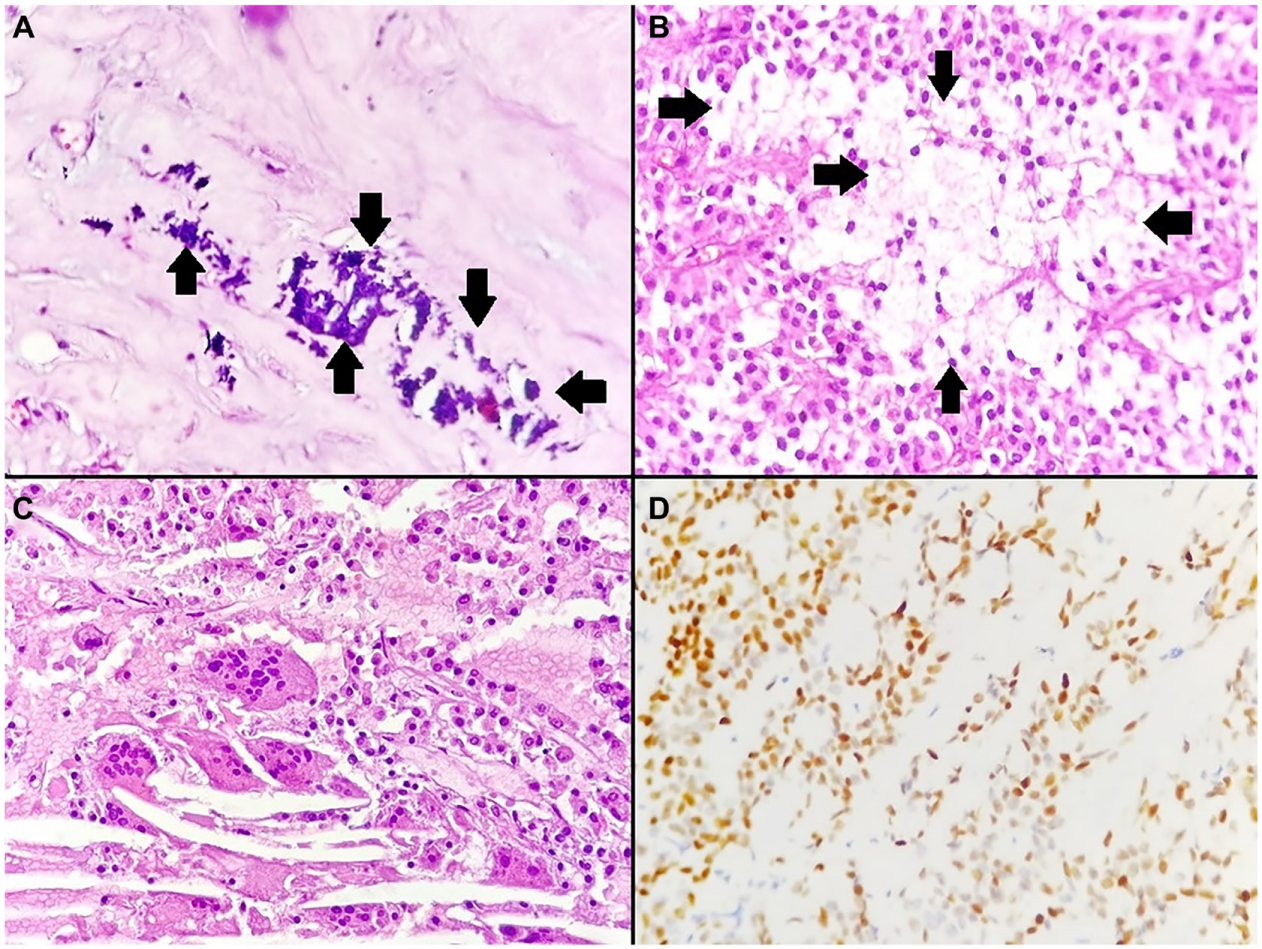
Histopathological and immunohistochemical features of the solid pseudopapillary neoplasm. (**A**) Basophilic calcifications within hyalinized fibrous areas (arrows) (H&E, ×400). (**B**) Tumor with focal infiltration by foamy histiocytes (arrows) (H&E, ×400). (**C**) Cholesterol clefts accompanied by multinucleated giant cells (H&E, ×400). (**D**) Tumor cells showing positive nuclear immunostaining for β-catenin (immunohistochemistry, ×400).

Gross examination revealed a well-encapsulated solid–cystic mass measuring 12.5 × 9 × 8 cm and weighing 656 g. The cut surface was heterogeneous, with areas of cystic degeneration, hemorrhage, and coarse calcifications (Figure 2). Histological examination showed a combination of solid and pseudopapillary patterns. The solid areas were composed of uniform tumor cells interspersed with numerous capillary-sized blood vessels (Figure 3). The stroma exhibited variable degrees of hyalinization and several degenerative changes. These included hemorrhage, calcifications (Figure 4A), infiltration by foamy histiocytes (Figure 4B), and cholesterol clefts (Figure 4C). Tumor cells showed moderate eosinophilic cytoplasm with intracytoplasmic hyaline globules, and mitotic figures were rare. Immunohistochemistry demonstrated nuclear positivity for β-catenin (Figure 4D) and cluster of differentiation 10 (CD10), confirming the diagnosis of SPN.

### Postoperative course and follow-up

The postoperative course was uneventful. The patient recovered well and was discharged on postoperative day seven. She has been under follow-up for five months, with no complications or evidence of recurrence detected.

## DISCUSSION

SPN is a rare pancreatic tumor. It represents approximately 5% of cystic pancreatic lesions [4]. This tumor predominantly affects young women, with a mean diagnostic age of 28 years and a female-to-male ratio of nearly 10:1 [5, 6]. Our case is consistent with this demographic profile, as it occurred in a 31-year-old woman. Most patients with SPN present with nonspecific abdominal symptoms such as pain, discomfort, or a palpable mass. However, up to 38% of tumors are discovered incidentally [7, 8]. Our patient presented with a two-year history of right upper abdominal pain, which worsened progressively. This course is typical of the indolent but symptomatic nature of SPN. Importantly, tumor markers such as CEA and CA19-9 were within normal limits, consistent with prior reports [7]. On CT, SPNs usually appear as well-encapsulated, heterogeneous masses with mixed solid and cystic components. Peripheral calcifications may also be present [9–11]. In our patient, CT showed a large lobulated pancreatic head mass (11.6 × 8.5 × 7.2 cm). It had solid, cystic, and unusually coarse calcified areas. This finding is rare and can mimic mucinous cystic neoplasms or neuroendocrine tumors. Despite its size and proximity to the duodenum and major vessels, there was no radiologic invasion. This reflects the characteristic displacing rather than infiltrative growth of SPN [9]. MRI confirmed the mixed solid–cystic architecture. Solid portions were iso- to hypointense on T1 and mildly hyperintense on T2. Cystic areas were bright on T2. Hemorrhagic degeneration appeared hyperintense on T1 sequences, a classic feature of SPN [10, 11]. There was no biliary or pancreatic ductal dilatation. This absence, despite the tumor arising in the pancreatic head, further supports the diagnosis. The presence of extensive, coarse calcification is a rare but key radiological feature of SPN. It helps narrow the differential diagnosis, which includes other pancreatic tumors. Importantly, within SPN, calcification does not appear to correlate with a more aggressive clinical course or worse prognosis [12]. Gross examination revealed a large encapsulated mass. The cut surface showed hemorrhage, cystic degeneration, and coarse calcifications. These features mirror the classic spectrum of degenerative changes seen in SPN [13]. Microscopically, the tumor displayed the classic biphasic pattern with solid and pseudopapillary areas. It also showed delicate capillary networks and several degenerative stromal changes. Immunohistochemical staining confirmed nuclear β-catenin and CD10 positivity. This is consistent with the established immunoprofile of SPN and critical for distinguishing it from morphologic mimics [14]. This staining pattern is a direct consequence of the distinct molecular pathogenesis of SPN. The hallmark genetic alteration is a somatic activating mutation in the CTNNB1 gene, which encodes β-catenin. This mutation leads to constitutive activation of the Wnt/β-catenin signaling pathway, resulting in nuclear β-catenin accumulation. These molecular events are considered the primary drivers of tumorigenesis in SPN. Importantly, other recurrent driver mutations typical of other pancreatic tumors are generally absent [15]. Surgical resection is the treatment of choice for SPN and generally offers excellent outcomes [16]. In our patient, a Whipple procedure was performed. This was given the tumor’s pancreatic head location and close relation to the duodenum and vascular structures. Although minimally invasive approaches are increasingly used, the size and calcified nature of the tumor made an open pancreaticoduodenectomy the most appropriate strategy [16, 17]. The prognosis of SPN is favorable, with cure rates exceeding 95% after complete resection [18]. In a systematic review of 2,158 patients, recurrence occurred in only 4.4% and disease-specific mortality in 1.5% [6]. Aggressive behavior has been associated with certain features [18, 19]. Our case showed no such features. Despite its large size and extensive calcifications, the tumor demonstrated low mitotic activity and no evidence of invasion. At five months of follow-up, the patient remains free of recurrence. Our case adds to the literature by documenting a giant calcified SPN of the pancreatic head in a young woman, successfully treated with pancreaticoduodenectomy. The extensive calcification represents an unusual finding that can complicate radiological diagnosis. Moreover, the tumor’s close proximity to major structures, yet absence of invasion, illustrates the paradoxical behavior of SPN. Our case highlights the importance of correlating imaging with histopathology and immunohistochemistry to secure diagnosis and ensure curative surgical management.

## CONCLUSIONS

SPN of the pancreas is a rare, low-grade tumor. Its variable imaging features pose diagnostic challenges. The presence of extensive coarse calcification an uncommon finding further complicates radiological assessment and highlights SPN as a key differential diagnosis for calcified pancreatic masses in young women. Accurate diagnosis depends on integrating imaging, histopathology, and immunohistochemistry. Complete surgical resection remains the definitive treatment and yields an excellent prognosis, even in large, calcified cases. Although recurrence is rare, follow-up for at least five years is recommended. This case emphasizes the need for high clinical suspicion in young women with heterogeneous pancreatic masses and the critical role of multidisciplinary collaboration in management.
